# *Lactobacillus casei* Strain Shirota Ameliorates Dextran Sulfate Sodium-Induced Colitis in Mice by Increasing Taurine-Conjugated Bile Acids and Inhibiting NF-κB Signaling *via* Stabilization of Iκ*B*α

**DOI:** 10.3389/fnut.2022.816836

**Published:** 2022-04-21

**Authors:** Wing-Yan Wong, Brandon Dow Chan, Tung-Ting Sham, Magnolia Muk-Lan Lee, Chi-On Chan, Chung-Ting Chau, Daniel Kam-Wah Mok, Yiu-Wa Kwan, William Chi-Shing Tai

**Affiliations:** ^1^Department of Applied Biology and Chemical Technology, The Hong Kong Polytechnic University, Hung Hom, Hong Kong SAR, China; ^2^The Laboratory for Probiotic and Prebiotic Research in Human Health, The Hong Kong Polytechnic University, Hung Hom, Hong Kong SAR, China; ^3^State Key Laboratory of Chinese Medicine and Molecular Pharmacology (Incubation), Shenzhen Research Institute, The Hong Kong Polytechnic University, Shenzhen, China; ^4^School of Biomedical Sciences, The Chinese University of Hong Kong, Sha Tin, Hong Kong SAR, China; ^5^Research Institute for Future Food, The Hong Kong Polytechnic University, Hung Hom, Hong Kong SAR, China

**Keywords:** probiotics, inflammatory bowel disease, *Lactobacillus casei* strain Shirota, gut microbiota, bile acids, DSS-induced acute colitis

## Abstract

Inflammatory bowel disease (IBD) is a chronic progressive intestinal inflammatory disease, characterized by an altered gut microbiota composition and accompanying alterations in circulatory bile acids. Increasing evidence supports the beneficial effect of probiotics intake on health. Introduction of probiotics to the intestines can modulate gut microbiota composition and in turn regulate the host immune system and modify the inflammatory response. Probiotics can also improve intestinal barrier function and exhibit a positive impact on host physiological and pathological conditions *via* gut microbiota-derived metabolites. Previous studies have demonstrated that *Lactobacillus casei* strain Shirota (LcS) treatment could inhibit clinical manifestation of colitis in dextran sulfate sodium (DSS)-induced mice, however, the underlying mechanisms remain unknown. In this study, we employed the DSS-induced acute colitis mouse model to investigate the anti-inflammatory effects of LcS and related mechanisms. Administration of LcS ameliorated the severity of DSS-induced colitis and enhanced intestinal integrity *via* induction of mucin-2 and occludin expression in colons. Fecal microbiota analysis showed that LcS increased the relative abundance of beneficial bacterial species in colitic mice, whereas the relative abundance of pathobionts was reduced. Additionally, LcS treatment modulated circulating bile acid profiles in colitic mice. In mice treated with LcS, we identified increased levels of primary taurine-conjugated bile acids, including taurocholic acid (TCA) and taurochenodeoxycholic acid (TCDCA). LcS treatment also increased the levels of secondary taurine-conjugated bile acids, including taurodeoxycholic acid (TDCA) and tauroursodeoxycholic acid (TUDCA). Moreover, LcS treatment exhibited a suppressive effect on the hydroxylated primary bile acids α-muricholic acid (α-MCA) and β-muricholic acid (β-MCA). We further demonstrated that LcS treatment suppressed the expression of pro-inflammatory mediators interferon-gamma (IFN-γ) and nitric oxide (NO), and increased the expression of the anti-inflammatory mediator interleukin-10 (IL-10) in colon tissues, potentially as a result of altered bile acid profiles. Mechanistically, we showed that LcS treatment suppressed the activation of nuclear factor-kappa B (NF-κB) signaling *via* stabilization of inhibitor of NF-κB alpha (IκBα). Altogether, we have demonstrated the therapeutic effects of LcS in DSS-induced colitis, providing new insights into its effect on bile acid metabolism and the related anti-inflammatory mechanisms. Our findings provide support for the application of LcS in the treatment of IBD.

## Introduction

Inflammatory bowel disease (IBD) describes a collection of conditions that share the common characteristic of chronic inflammation of the gastrointestinal tract, often presenting with non-specific clinical features such as abdominal pain, recurring or bloody diarrhea, weight loss, and fatigue (1). Over the past several decades, IBD has emerged as a challenge against public health worldwide. An estimated 6.8 million people suffer from IBD and an increasing trend in IBD incidence has been observed globally, especially in newly industrialized countries (2, 3).

While the exact cause of IBD is unknown, evidence suggests that a complex interplay of genetics, intestinal microbiota, external environment, immunity, and other factors are involved (4). A healthy gut microbiota ecosystem is considered necessary for the normal health of its host. Conversely, loss of microbial balance, also known as dysbiosis, is associated with dysfunction and disease. Local effects of dysbiosis are readily implicated in intestine-related diseases, including IBD (5). Although the microbiota is resilient, it can be altered within individuals by both internal and external stimuli. This microbiotal plasticity creates a distinct opportunity to reshape dysbiotic conditions associated with disease and guide individuals toward improved health. Hence, there is considerable interest in modulating the state and function of the gut microbiota to achieve preventive and/or therapeutic benefits in disease.

Probiotics can act to modulate the existing intestinal microbiota, produce mediators that can modify the inflammatory response and gastrointestinal function, improve intestinal barrier function, and alter the host immune response (6, 7). *Lactobacillus casei* strain Shirota (LcS) is a well-known probiotic strain that was first isolated in 1930, and in 2013 attained generally recognized as safe (GRAS) status from the USFDA. LcS has been reported to exhibit various beneficial effects on gastrointestinal function and disorders, including prevention of diarrhea (8), improved bowel movements (9), alleviating colitis symptoms (10), and has also been shown to demonstrate immunomodulatory properties (11, 12).

Additionally, increasing interest has been directed toward gut microbiota-derived metabolites, as they have been proposed to exhibit diverse effects on host physiology and pathology (13). Bile acids are cholesterol-derived acids produced in the liver, originally recognized for their function in emulsification of dietary lipids and facilitation of their absorption. However, emerging evidence has shown that bile acids can act as pleiotropic signaling metabolites that participate in multiple metabolic and inflammatory pathways through dynamic interaction with the gut microbiota (14). It has been proposed that the gut microbiome directly impacts the composition of circulating bile acids in the host, and that alteration of the gut microbiota can lead to changes in bile acid levels (15). In IBD, patients exhibit dysbiosis accompanied by altered circulatory bile acid composition (16, 17), and studies have shown that treatment of colitis mice with bile acids could ameliorate the dysbiotic gut microbiome (18). This relationship between the host and their gut microbiota suggests that probiotics could have a regulatory influence on host bile acid metabolism and immunity.

As *Lactobacillus* species are involved in bile acid metabolism (14, 19), we hypothesized that in addition to alteration of the gut microbiota, LcS could exhibit anti-inflammatory effects through modulation of bile acid metabolism. In this study, using the dextran sulfate sodium (DSS)-induced acute colitis mouse model, we aimed to demonstrate the anti-inflammatory effects of LcS and elucidate the underlying molecular mechanisms, including its potential effects on bile acid metabolism.

## Materials and Methods

### Bacterial Strain and Bacterial Culture

*Lactobacillus casei* strain Shirota (LcS) was provided as a gift from Prof. Y.W. Kwan of The Chinese University of Hong Kong. To prepare the starter culture, the strain was propagated in sterile MRS medium (10 ml) anaerobically at room temperature (without shaking) until an OD_600_ of 1.2 was reached. 1 ml of starter culture was then transferred to MRS broth (1.1 L) and cultured until an OD_600_ of 1.2 was reached. Subsequently, the bacterial culture was centrifuged (8,000 rpm, 15°C, 10 min), washed with distilled water, and the supernatant discarded. The bacterial pellet was collected and re-suspended in PBS (with 10% glycerol, vol./vol.); cell count was adjusted to 1 × 10^11^ CFU/ml, and stored in a −80°C freezer. Before use, the final solution was enumerated on MRS agar to confirm the dose administered to the mice.

### Animal Studies

Male and female wild-type C57BL/6J mice were purchased from the Jackson Laboratory (Bar Harbor, ME, United States) and mated to maintain an inbred breeding colony at The Hong Kong Polytechnic University Centralised Animal Facilities. Mice were maintained in a barrier-sustained animal house, air-conditioned at 20 ± 2°C and humidity at 55 ± 10%, under a 12 h light/dark cycle. Standard diet and water were available *ad libitum*. All animal experiments were approved by the Animal Subjects Ethics Sub-Committee (ASESC) of The Hong Kong Polytechnic University and conducted in accordance with the Institutional Guidelines and Animal Ordinance of the Department of Health, Hong Kong SAR, China.

### Dextran Sulfate Sodium-Induced Acute Colitis and Treatment

The acute colitis animal study was performed as previously described, with minor modifications (20). Briefly, 8-week-old male wild type C57BL/6J mice were randomly assigned to three groups (*n* = 15 per group): uninduced control, colitis, and colitis with LcS treatment. Acute colitis was induced in mice by supplying 2.5% w/v DSS (reagent grade; 36,000–50,000 Da; MP Biomedicals, Solon, OH, United States) in their drinking water for 7 consecutive days, during which the mice were treated daily *via* oral gavage with vehicle or LcS (1 × 10^10^ CFU). Body weight, and food and water consumption were recorded daily. Disease activity index (DAI) was determined as described previously (21), calculated as the sum of the following parameters: weight loss (0 points = No weight loss, 1 point = 1–5% weight loss, 2 points = 6–10% weight loss, 3 points = 11–20% weight loss, and 4 points = > 20% weight loss, compared with initial body weight); stool consistency (0 points = normal, 2 points = loose, and 4 points = diarrhea); and fecal blood (0 points = normal, 2 points = slight blood in feces, and 4 points = gross blood in feces). At the end of the treatment period, mice were sacrificed, colons removed, and their lengths measured. Cecal contents were collected and stored at −80°C until analysis. Colons were washed with PBS and were either cultured, snap frozen, or fixed for further assessment.

### Colon Tissue Culture

Colon tissue cultures were conducted as previously described (21). Briefly, 1 cm sections of colon tissue from each mouse were cultured in RPMI 1640 culture medium supplemented with 10% FBS and 50U P/S for 24 h at 37°C. Supernatants were collected after centrifugation and stored at −80°C until use.

### Griess Assay

Supernatants of colon tissue cultures were collected, and nitric oxide (NO) levels were quantified using the Griess Reagent System according to manufacturer’s instructions (Promega, Madison, WI, United States). Briefly, 50 μl of supernatant or nitrite standards were added to 96-well plate, followed by 50 μl sulfanilamide solution and 50 μl N-1-naphthyl ethylenediamine dihydrochloride (NED) solution, with a 10 min incubation period following the addition of each. Absorbance at 550 nm was measured using a Clariostar Monochromator Microplate Reader (BMG Labtech, Germany), and nitrite concentrations were calculated by comparison to the standard curve.

### Western Blotting

Western blot analysis was performed as described previously (21). Briefly, colon tissues were lysed in RIPA buffer and then centrifuged to remove cellular debris. Samples were electrophoresed through SDS-PAGE gels and transferred onto PVDF membranes (Bio-Rad, United States). The blots were then blocked in 5% non-fat skim milk in TBS-Tween 20 (TBST) buffer and probed using the following antibodies in 5% BSA in TBST buffer overnight: Hsp70, Hsp90α/β, β-actin (Santa Cruz Biotechnology, Santa Cruz, CA, United States), iNOS (BD Biosciences, CA, United States), IKKα, IKKβ, phospho-IKKα/β, IκBα, phospho-IκBα, NF-κB, and phospho-NF-κB (Cell Signaling Technology, Danvers, MA, United States). Blots were then incubated with the corresponding goat anti-rabbit, donkey anti-goat (Santa Cruz Biotechnology), and goat anti-mouse (Life Technologies) HRP-conjugated secondary antibodies. Protein bands were visualized using Clarity ECL Western blotting substrates (Bio-Rad). Images were obtained using a ChemiDoc Imaging System (Bio-Rad) and protein expression was analyzed using Image Lab software (Bio-Rad).

### Assessment of Colonic Gene Expression

Total RNA was isolated from 20 mg mouse colon tissue using the E.Z.N.A. Total RNA Kit I (Omega Bio-tek, United States), according to manufacturer’s instructions. Concentration and purity of extracted RNA were measured using a Nanodrop One spectrophotometer (Thermo Scientific, United States). First strand cDNA synthesis was carried out from 1 μg RNA using SuperScript VILO MasterMix (Invitrogen, United States). Relative gene expression in colon samples was assessed by quantitative PCR (qPCR). PCR reaction mixtures contained 10 μl of 2 × SYBR Green Master Mix (Applied Biosystems, United States), 400 nM of each forward and reverse primers, and 2 μl diluted cDNA sample. The sequences of primers used for qPCR are listed in Table 1. Amplification was performed using the QuantStudio 7 system (Applied Biosystems) at the following conditions: 2 min at 50°C, 10 min at 95°C, followed by 45 cycles of 15 s at 95°C and 1 min at 60°C. Relative expression was calculated using the 2^–ΔΔ*CT*^ method, with β-actin as a reference gene.

**TABLE 1:** Primers used in qPCR analysis.

Gene	Forward primer (5′–3′)	Reverse primer (5′–3′)
Muc2	GCTGACGAGTGGTTGGTGAATG	GATGAGGTGGCAGACAGGAGAC
IFN-γ	TCAAGTGGCATAGATGTGGAAGAA	TGGCTCTGCAGGATTTTCATG
IL-10	AACATACTGCTAACCGACTCCT	CTGCCTTGCTCTTATTTTCACA
β-actin	GACAGGATGCAGAAGGAGATTACT	TGATCCACATCTGCTGGAAGGT

**Target**	**Forward primer (5′–3′)**	**Reverse primer (5′–3′)**

*Prevotellaceae*	CCAGCCAAGTAGCGTGCA	TGGACCTTCCGTATTACC
*Bifidobacterium*	GCGTGCTTAACACATGCAAGTC	CACCCGTTTCCAGGAGCTATT
*Lactobacillus*	TGGAAACAGRTGCTAATACCG	GTCCATTGTGGAAGATTCCC
*Clostridium cluster IV*	GCACAAGCAGTGGAGT	CTTCCTCCGTTTTGTCAA
*Lactobacillus casei*	ACCGCATGGTTCTTGGC	CCGACAACAGTTACTCTGCC
Total bacteria	ACTCCTACGGGAGGCAGCAGT	GTATTACCGCGGCTGCTGGCAC

### Assessment of Cecal Bacterial Abundance

DNA was extracted from approximately 250 mg mouse cecum contents using the QIAamp PowerFecal DNA Kit (Qiagen, Hilden, Germany), according to manufacturer’s instructions. Concentration and purity of extracted DNA were measured using a Nanodrop One spectrophotometer. Relative bacterial abundance in cecal samples was assessed by qPCR. PCR reaction mixtures contained 10 μl of 2 × SYBR Green Master Mix, 400 nM of each forward and reverse primers, and 2 μl (5 ng) DNA sample. The sequences of primers used for qPCR are listed in Table 1. Amplification was performed using the QuantStudio 7 system with the programme described above. Relative expression was calculated using the 2^–ΔΔ*CT*^ method. Total bacteria, the expression of which was unaffected by treatment, was used as a reference.

### Bile Acid Assessment

Acetonitrile and methanol (HPLC grade) were purchased from Duksan (Ansan-si, Gyeonggi-do, South Korea). Distilled water was purified using a Milli-Q water purification system (Millipore, Bedford, MA, United States). Formic acid, hyodeoxycholic acid, ursodeoxycholic acid, sodium chenodeoxycholate, sodium tauroursodeoxycholate, and sodium taurochenodeoxycholate were purchased from Sigma-Aldrich (St. Louis, MO, United States). α-Muricholic acid, deoxycholic acid, and taurodeoxycholic acid (TDCA) were purchased from Santa Cruz Biotechnology (Dallas, TX, United States). Glycoursodeoxycholic acid, cholic acid-2,2,4,4-D4, glycoursodeoxycholic acid-2,2,4,4-D4, deoxycholic acid-2,2,4,4-D4, and ursodeoxycholic acid-2,2,4,4-D4 were purchased from Cambridge Isotope Laboratories (Tewksbury, MA, United States). Sodium glycocholate hydrate was purchased from Acros Organics (Morris Plains, NJ, United States). Cholic acid, β-muricholic acid, glycodeoxycholic acid, glycohyodeoxycholic acid, taurocholic acid, sodium glycochenodeoxycholate, taurohyodeoxycholic acid, lithocholic acid, glycocholic acid-2,2,4,4-D4, and glycochenodeoxycholic acid-2,2,4,4-D4 were obtained from Steraloids (Newport, RI, United States).

Bile acids, conjugated bile acids, and internal standards (Supplementary Table 1) were dissolved individually in methanol at a concentration of 1,000 ppm. The bile acids and conjugated bile acids were mixed as a stock mixture (50 ppm). 14 calibration standard working solutions containing the bile acids and conjugated bile acids at 0.0152 ppb–2 ppm were prepared by serial dilution (two-fold) of the stock mixture with methanol. An internal standard mixture was prepared by mixing each of ursodeoxycholic acid-D4, deoxycholic acid-D4, cholic acid-D4, glycoursodeoxycholic acid-D4, glycochenodeoxycholic acid-D4, and glycocholic acid-D4 with methanol to a final concentration of 250 ppb. 150 μl of each calibration standard working solution was mixed with 50 μl of the internal standard mixture at the same concentration as study samples. Standards were injected into the ultra-performance liquid chromatography (UPLC)-MS/MS at the same conditions as study samples.

The method of serum preparation and UPLC-MS/MS were optimized based on a validated method (22). 50 μL serum from each sample was deproteinated with 100 μl cold methanol and 50 μl of the cold internal standard mixture (diluted to the same concentration as the calibration curve solution). Samples were vortexed for 30 s and deproteinated at −20°C for 1 h prior to centrifugation at 20,000 rpm at 4°C for 15 min. 80 μl supernatant was transferred into an insert of an amber HPLC vial for UPLC-MS/MS.

A 3 μL aliquot was injected into a SCIEX ExionLC AD UPLC system coupled to a SCIEX quadrupole-ion trap (QTRAP) 6500^+^ system. The order of injection for all samples was randomized. UPLC separation was performed on a reverse-phase Waters ACQUITY UPLC HSS T3 column (2.1 mm × 100 mm, 1.8 μm) with HSS T3 guard column (2.1 mm × 5 mm, 1.8 μm). The mobile phase consisted of combinations of A (0.1% formic acid in water, v/v) and B (0.1% formic acid in acetonitrile, v/v) at a flow rate of 0.3 ml/min with elution gradient as follows: 0 min, 35% B; 3 min, 40% B; 9 min, 45% B; 12–15 min, 95% B. A 2.5-min post-run time was set to fully equilibrate the column. Column and sample chamber temperature were 45 and 8°C, respectively. The mass-spectrometric parameters were set as follows: ionspray voltage, −4500 V in ESI negative ionization mode; curtain gas, 20 psi; desolvation temperature, 500°C; ion source nebulizer gas (GS1) and heater gas (GS2), 60 psi and 50 psi; collision gas (nitrogen gas), low. The ions were monitored in Multiple Reaction Monitoring (MRM) mode (Supplementary Table 1).

Quantitation tools within SCIEX OS Software (Version 1.6.1.29803) were employed to process all UPLC-MS/MS data, draw the calibration curves, and calculate the concentration of standards and samples. Calibration curves were analyzed using a linear fit with a 1/x weighting if there was a large dynamic range of the concentrations.

### Immunohistochemical Analysis

Colon sections were fixed in formalin solution and embedded in paraffin. Hematoxylin and eosin (H&E) and phosphorylated NF-κB staining were conducted as previously described (23). Briefly, paraffin-embedded tissues were cut into 4 μm sections, and deparaffinized and rehydrated using xylene and alcohol. Rehydrated sections were either stained with hematoxylin and eosin or phosphorylated NF-κB using the LSAB2 System-HRP kit (Dako, Agilent, Santa Clara, CA, United States) according to manufacturer’s instructions. In brief, tissue sections were blocked using peroxidase blocking solution and were then incubated with phosphorylated NF-κB primary antibody (Santa Cruz Biotechnology) at room temperature. After subsequent incubation with biotinylated streptavidin-HRP solution and enzyme substrate, the sections were counterstained with hematoxylin, dehydrated, mounted and examined under a Leica DMI 3000B inverted microscope (Leica Microsystems, Wetzlar, Germany).

For CD45 immunofluorescence staining, tissue sections were stained with CD45 primary antibody (Abcam, Cambridge, MA, United States) at room temperature after blocking with peroxidase blocking solution. Sections were washed and stained with goat anti-rabbit secondary antibody conjugated with Alexa Fluor 594 (Abcam) at room temperature. Then, the sections were counterstained with DAPI, mounted, and examined under a microscope.

Alcian blue staining was conducted using Alcian blue/nuclear fast red. Briefly, 4 μm tissue sections were deparaffinized and rehydrated, followed by staining in Alcian blue solution and subsequent counter-staining in nuclear fast red solution. The tissue sections were then mounted and examined under microscopy.

### Statistical Analysis

Data are presented as mean ± SEM. Normality of distribution and homogeneity of variances were confirmed using the Shapiro-Wilk and Brown–Forsythe tests, respectively, and differences between groups were assessed using one way or two way ANOVA when appropriate, with Tukey’s *post-hoc* test. *P*-values < 0.05 were considered statistically significant.

## Results

### *Lactobacillus casei* Strain Shirota Reduced the Severity of Dextran Sulfate Sodium-Induced Acute Colitis

**FIGURE 1:**
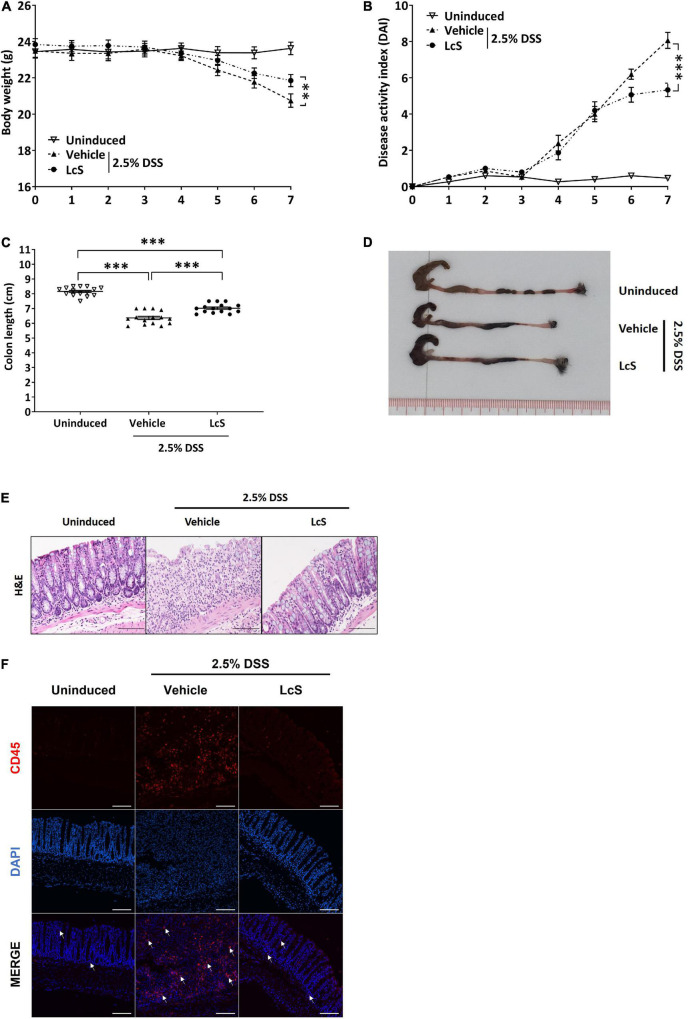
LcS attenuated the severity of acute colitis in DSS-induced mice. Acute colitis was induced in C57BL/6J mice *via* administration of 2.5% DSS in drinking water. During the induction period, mice were fed orally with vehicle or LcS. Mice fed with normal drinking water and vehicle served as an uninduced group. Mice **(A)** body weight and **(B)** DAI were monitored daily. At the end of the experiment, mice were terminated and **(C)** colon lengths were measured. **(D)** Representative photographs of mice colons. **(E)** Representative H&E staining images of mice colons. **(F)** Representative images of CD45 immunofluorescence staining (red) in mouse colons. White arrows indicate positive stain. Data are expressed as mean ± SEM; ^**^p < 0.01, ^***^p < 0.001. Scale bars: H&E, 50 μm; immunofluorescence staining, 25 μm.

We first evaluated the effect of LcS on colitis in DSS-induced mice. In comparison with uninduced mice, mice induced with 2.5% DSS showed a significant reduction in body weight and increased DAI scores (Figures 1A,B), indicating an increase in the severity of colitis after DSS induction. Under DSS induction, LcS-treated mice showed significant improvements in body weight and a reduction in DAI scores when compared with vehicle-treated mice. Further, we found that LcS treatment significantly increased colon length in DSS-induced mice (Figures 1C,D). We next examined histological changes in the colon after treatment with LcS. Compared to uninduced mice, DSS-induced mice showed severe pathological changes in their colons, including epithelial erosion, loss of crypts, and extensive infiltration of CD45^+^ leukocytes, typical features of colitis. Notably, LcS treatment ameliorated these pathological changes associated with colitis in mice (Figures 1E,F). Altogether, our results demonstrated that LcS treatment effectively alleviated disease manifestations in acute colitis.

### *Lactobacillus casei* Strain Shirota Treatment Ameliorated Dextran Sulfate Sodium-Induced Damage to Intestinal Integrity Through Colonic Induction of Mucin-2 and Occludin Expression

**FIGURE 2:**
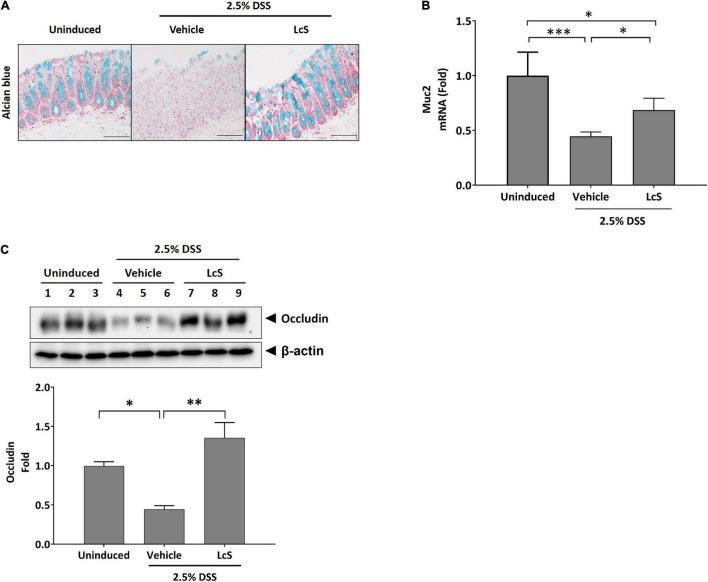
LcS restored measures of intestinal integrity in DSS-induced mice. **(A)** Representative Alcian blue staining images and **(B)** Muc2 gene expression levels in mouse colons. Gene expression levels were normalized to uninduced mice. **(C)** Representative Western blot images of occludin in mouse colons. Protein expression levels were normalized to uninduced mice. Data are expressed as mean ± SEM; *p < 0.05, ^**^p < 0.01, ^***^p < 0.001. Scale bars, 50 μm.

As treatment with LcS ameliorated the destructive effect of DSS on the colon structures of induced mice, we thus investigated whether LcS treatment could promote physical barrier maintenance during acute colitis. DSS-induced mice exhibited significant mucin depletion in their colons (Figure 2A). When mice were treated with LcS, increased Alcian blue staining in colons was observed, suggesting that LcS treatment could ameliorate damage to the mucus layer resulting from colitis. To confirm the effect of LcS on mucin production, we also quantified colon mRNA levels of mucin-2 (Muc2), the transcription factor for mucin production. Expression of Muc2 in the colons of DSS-induced mice was suppressed when compared with uninduced mice, however, treatment with LcS significantly increased Muc2 expression, suggesting that LcS could increase the production of Muc2 at the transcript level (Figure 2B).

We also evaluated the effect of LcS on the expression of the tight junction protein occludin in mouse colons. As shown in Figure 2C, DSS-induced mice exhibited reduced expression of occludin in colons, whereas upregulation of the protein was observed after treatment with LcS. Our findings indicate that LcS treatment could maintain intestinal integrity in acute colitis *via* preservation of the mucus layer and tight junctions.

### *Lactobacillus casei* Strain Shirota Treatment Altered the Gut Microbiota Composition of Dextran Sulfate Sodium-Induced Mice

**FIGURE 3:**
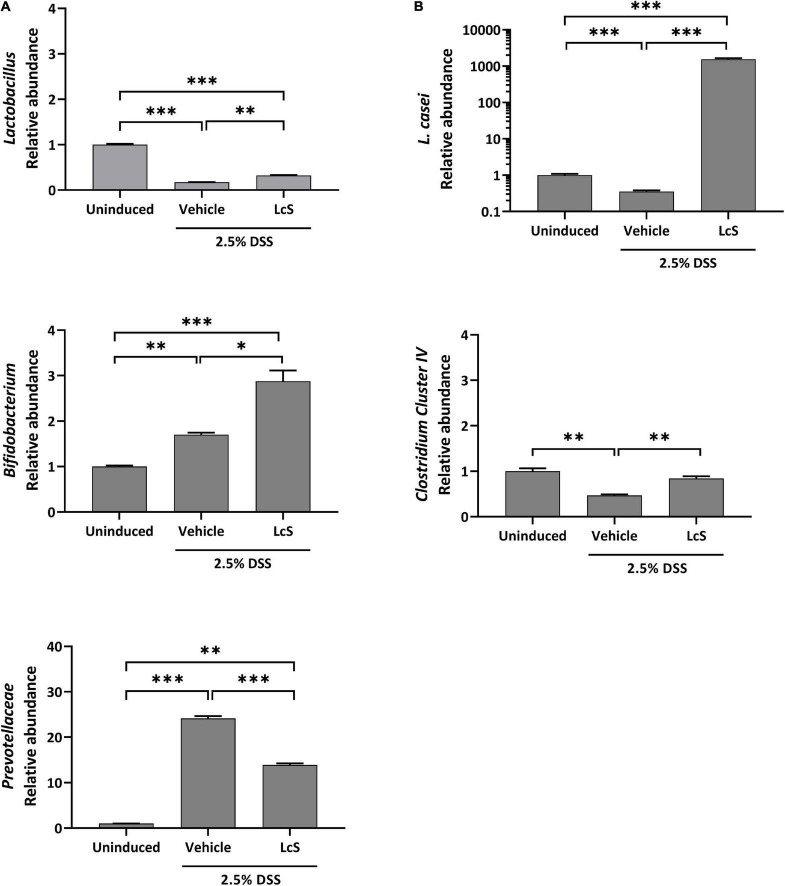
LcS modulated gut microbiota in DSS-induced mice. Relative abundance of **(A)** *Lactobacillus*, *Bifidobacterium*, and *Prevotellaceae*, and **(B)** *L. casei* and *Clostridium cluster IV* in mice cecal contents. Bacterial abundances were normalized to DSS-induced mice. Data are expressed as mean ± SEM; *p < 0.05, ^**^p < 0.01, ^***^ *p* < 0.001.

Next, we investigated the effect of LcS on the composition of the gut microbiota. We selected five bacterial targets that have been demonstrated to have altered abundances in colitis, including the family *Prevotellaceae*, the genera *Lactobacillus*, *Bifidobacterium*, and *Clostridium cluster IV*, and the species *Lactobacillus casei* (*L. casei*) (24–26), and examined their relative abundances in mouse cecal contents. Increased relative abundance of the pathobiont *Prevotellaceae* was observed in DSS-induced mice when compared with uninduced mice, whereas LcS treatment significantly reduced their relative abundance. In contrast, LcS treatment increased the relative abundance of the beneficial bacteria *Lactobacillus*, *Bifidobacterium*, and *Clostridium cluster IV* (Figure 3). In addition, LcS treatment substantially enhanced the relative abundance of *L. casei* in DSS-induced mice, which was significantly reduced in DSS-induced mice when compared with uninduced control (Figure 3B). These results suggest that LcS treatment increased the relative abundance of beneficial bacteria and reduced the relative abundance of pathogenic bacteria, potentially contributing to disease improvement.

### *Lactobacillus casei* Strain Shirota Affected Bile Acid Metabolism and Suppressed the Pro-inflammatory Response

**FIGURE 4:**
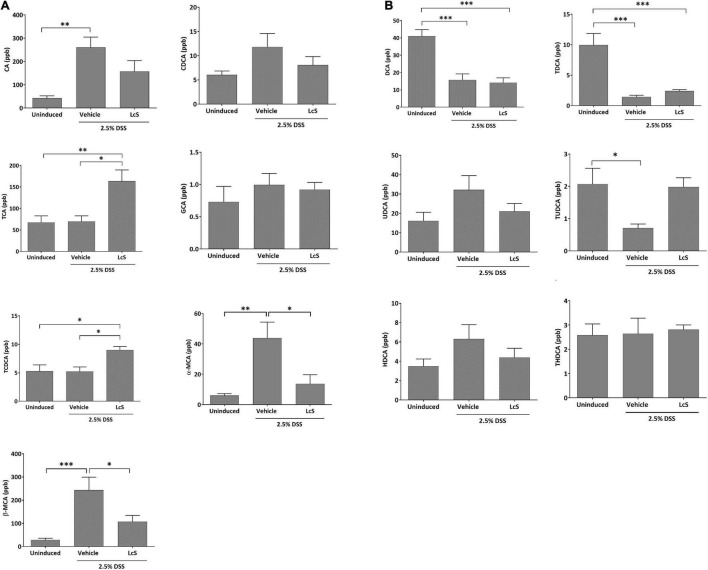
LcS altered bile acid metabolism in DSS-induced mice. Levels of **(A)** CA, CDCA, TCA, GCA, TCDCA, α-MCA, and β-MCA, and **(B)** DCA, TDCA, UDCA, TUDCA, HDCA, and THDCA in mouse plasma. Data are expressed as mean ± SEM; *p < 0.05, ^**^p < 0.01, ^***^p < 0.001.

To explore the effect of LcS on bile acid metabolism, we evaluated and compared bile acid profiles in the plasma of DSS-induced mice. As shown in Figure 4, DSS-induced mice treated with LcS showed significant increases in circulatory taurine-conjugated bile acids. When compared with vehicle-treated mice, mice treated with LcS displayed increased levels of the primary taurine-conjugated bile acids taurocholic acid (TCA) and taurochenodeoxycholic acid (TCDCA), and decreased DSS-induced levels of the hydroxylated primary bile acids α-muricholic acid (α-MCA) and β-muricholic acid (β-MCA) (Figure 4A). LcS treatment also increased levels of the secondary taurine-conjugated bile acids taurodeoxycholic acid (TDCA) and tauroursodeoxycholic acid (TUDCA) in DSS-induced mice (Figure 4B).

**FIGURE 5:**
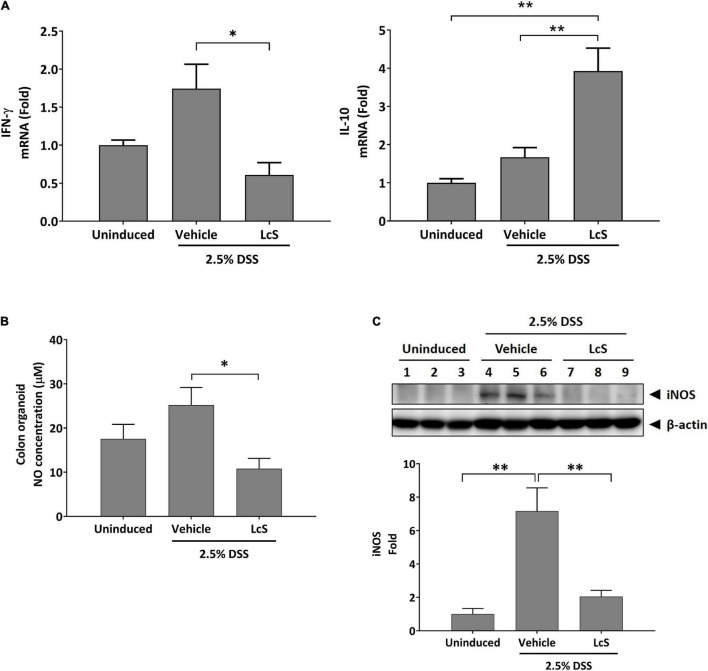
LcS modulated inflammatory mediator production. Gene expression levels of **(A)** IFN-γ and IL-10 in mouse colons. Expression levels of the genes were normalized to uninduced mice. **(B)** NO production levels in mouse colon cultures. **(C)** Representative Western blot images of iNOS in mouse colons. Protein expression levels were normalized to uninduced mice. Data are expressed as mean ± SEM; *p < 0.05, ^**^p < 0.01.

To assess whether the increased levels of taurine-conjugated bile acids in DSS-induced mice treated with LcS resulted in an anti-inflammatory response, we examined inflammatory mediators IFN-γ and IL-10 mRNA expression in mouse colons. As shown in Figure 5A, DSS-induced mice treated with LcS exhibited significant downregulation of IFN-γ and upregulation of IL-10 in colon tissues when compared with vehicle-treated mice.

We next assessed whether the upregulation of IL-10 in LcS-treated mice would result in modulation of NO production. As shown in Figure 5B, NO production in the colons of DSS-induced mice was significantly reduced after treatment with LcS. In addition, the protein expression of nitric oxide synthase (iNOS), the upstream modulator of NO production, was also downregulated after treatment with LcS in DSS-induced mice (Figure 5C). Collectively, our results showed that LcS treatment exhibited anti-inflammatory effects *via* increasing taurine-conjugated bile acids.

### *Lactobacillus casei* Strain Shirota Suppressed Activation of NF-κB Signaling in Colons

**FIGURE 6:**
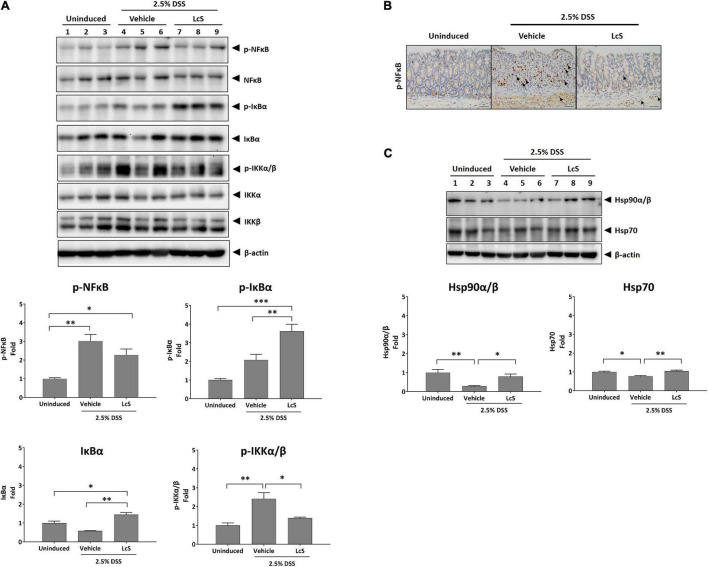
LcS suppressed NF-κB signaling. Western blot analysis of **(A)** IKKα/β, IκBα, NF-κB, and **(C)** Hsp90α/β and Hsp70 in mouse colons. Expression levels of the proteins were normalized to uninduced mice. **(B)** Representative immunohistochemical staining images of phosphorylated NF-κB in mouse colons. Arrows indicate colocalization of phosphorylated NF-κB with nuclei. Data are expressed as mean ± SEM; *p < 0.05, ^**^p < 0.01, ^***^p < 0.001. Scale bars, 25 μm.

Next, we sought to explore the effects of LcS on the NF-κB signaling pathway. As shown in Figure 6A, increased expression of phosphorylated IKKα/β, IκBα, and NF-κB were observed in the colons of DSS-induced mice, suggesting activation of the NF-κB signaling pathway. In comparison, expression of phosphorylated IKKα/β and NF-κB were downregulated in LcS-treated mice. Notably, we identified a significant increase in the expression of phosphorylated IκBα in LcS-treated mice colons, accompanied by a significant increase in IκBα. These results suggest LcS may inhibit proteasomal degradation of IκBα in the colon by stabilizing IκBα, inhibiting its dissociation from NF-κB and the activation of NF-κB.

Although mild reduction in the expression of NF-κB was observed in mice treated with LcS, we reasoned that the stabilization of IκBα might inhibit nuclear translocation of NF-κB rather than suppress its phosphorylation. To confirm the effect of LcS on translocation of NF-κB, we performed immunohistochemical analysis of phosphorylated NF-κB in mouse colons. In DSS-induced mice, we observed an increased number of cells expressing phosphorylated NF-κB colocalized with nuclei, whereas in mice treated with LcS, there was a reduction in the number of cells expressing phosphorylated NF-κB colocalized with nuclei (Figure 6B).

In addition, we also confirmed a significant increase in expression of the heat shock proteins Hsp90α/β and Hsp70 in LcS-treated mice when compared with DSS-induced mice (Figure 6C), further suggesting an inhibitory effect of LcS on proteasomal degradation. Collectively, these results demonstrated that LcS suppressed the activation of NF-κB signaling *via* stabilization of IκBα.

## Discussion

In this study, we have shown that LcS could modulate bile acid metabolism and exhibit anti-inflammatory effects in a DSS-induced acute colitis mouse model. We first demonstrated that LcS treatment could strengthen intestinal integrity *via* increasing the expression of occludin and Muc2 in DSS-induced mouse colons. The tight junction proteins and the mucus layer comprise the first line of defense against commensal bacteria and pathogens in the colon. Loss of this physical barrier can lead to infiltration of pathogens, contributing to the pathological process of IBD and resulting in inflammation (27, 28). Occludin is one of the major intestinal tight junction proteins involved in maintaining the structural integrity of the colon (29, 30), and is observed to be downregulated during colitis (31). In addition, deficiency in Muc2 expression leads to spontaneous development of colitis in mice, and diminished mucus production has been observed in IBD patients (32, 33). Furthermore, mucin expression has been shown to be inversely correlated with IBD severity and inflammation levels in the gut (34). As LcS treatment can restore tight junction protein expression and stimulate mucus production, it can aid re-establishment of the intestinal barrier and potentially reduce pathogen infiltration and diminish the inflammatory response in IBD conditions.

LcS treatment also altered the gut microbiota composition of DSS-induced mice. It has been shown that the abundance of pathobionts were increased and beneficial bacteria decreased in the feces of IBD patients and colitis mice (24–26), suggesting the importance of bacteria in the pathogenesis of IBD. We demonstrated that LcS treatment increased not only the levels of *L. casei*, but also the abundance of other beneficial bacteria including *Lactobacillus*, *Bifidobacterium*, and *Clostridium cluster IV*, while the abundance of the pathobiont *Prevotellaceae* was decreased. These results suggested administration of LcS not only altered the abundance of specific bacteria, but could also ameliorate the dysbiotic condition in colitis.

In addition to the gut microbiota composition, LcS also altered the bile acid profile in DSS-induced mice. After LcS treatment, we identified increases in levels of the circulatory taurine-conjugated bile acids TCA, TCDCA, TDCA, and TUDCA, and significant reductions in the hydroxylated primary bile acids α-MCA and β-MCA. As taurine-conjugated bile acids have been proposed to exhibit anti-inflammatory properties (35–38), the induction of increases in taurine-conjugated bile acids by LcS highlights its anti-inflammatory potential. Further, TCA and TCDCA have been identified as ligands of farnesoid X receptor (FXR) (39, 40), whereas α-MCA and β-MCA are FXR antagonists (41). Additionally, TDCA and TUDCA have been identified as agonists of G protein-coupled bile acid receptor 1 (GPBAR1) (42–44). FXR and GPBAR1 (also known as TGR5) are bile acid receptors expressed on monocytes and macrophages, and activation of these receptors have been shown to induce anti-inflammatory effects and protect against colitis in mice (45, 46). These results suggested that LcS treatment indeed modulates bile acid metabolism and is beneficial, as these molecules can interact with FXR and GPBAR1 receptors to exhibit anti-inflammatory effects. In addition, TUDCA has been shown to exhibit protective effects against colitis (18, 35, 38), suggesting further suppressive effects on the inflammatory response by metabolites induced by LcS. Thus, changes in bile acid metabolism resulting from LcS treatment regulated host immunity through different means.

We also found that LcS treatment could induce production of IL-10. IL-10 is an anti-inflammatory cytokine secreted by a broad range of immune cells including macrophages, dendritic cells, neutrophils, eosinophils, T-cells, and B-cells (47). IL-10 has been identified as a key suppressive mediator of IBD; various genome-wide association studies have shown that IL-10 and its receptor IL-10R play important protective roles in IBD (48–50). IL-10 deficient mice develop spontaneous colitis (51), and IL-10^–/–^ mice are an increasingly employed animal model for the study of IBD. In clinical studies, increased serum levels of IL-10 have been linked to the recovery phase in IBD patients (52), indicating the beneficial role it can play in IBD. In addition, activation of FXR and GPBAR1 in pro-inflammatory macrophages can induce polarization toward the anti-inflammatory M2 phenotype, which is characterized by upregulation of IL-10 and downregulation of IFN-γ, further suggesting the anti-inflammatory mechanism of LcS (45). Studies have also shown that IL-10 is involved in restoration of the intestinal epithelial barrier and suppression of NO production (53, 54), observations that are in line with our findings, where we identified increases in Muc2 and occludin expression and downregulation of iNOS and NO production after treatment with LcS.

**FIGURE 7:**
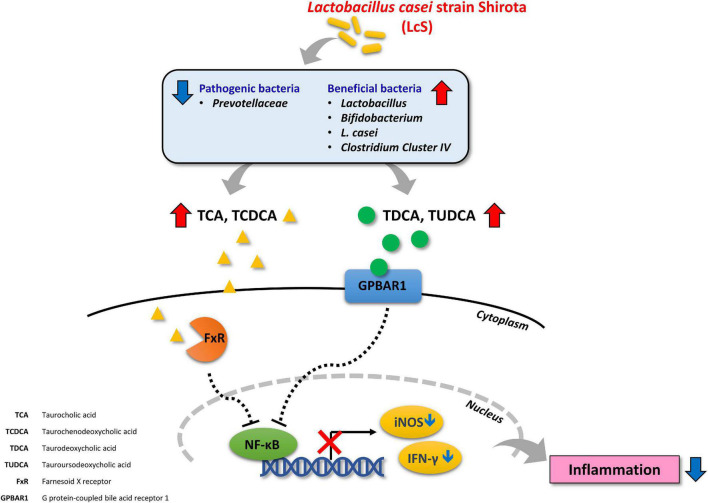
Overview of the proposed anti-inflammatory mechanism of LcS in IBD.

NF-κB signaling has been proposed as a major contributing pathway to the pathogenesis of IBD (55). Inhibition of proteasomal degradation and stabilization of phosphorylated IκBα maintains its inhibitory effect on NF-κB signaling (56, 57). In our results, we identified increased levels of p-IκBα in colon lysates from mice treated with LcS, and decreased expression of p-NF-κB, suggesting that LcS could potentially inhibit degradation of IκBα and the subsequent activation of NF-κB. This mechanism has also been implicated in the activity of other anti-IBD therapeutics, such as corticosteroids and anti-TNF-α antibodies. In addition, we identified increased expression of Hsp90α/β and Hsp70, implicating inhibition of proteasomal degradation in the mechanism of LcS, as proteasome inhibitors have been shown to be potent stimulators of heat-shock proteins (58). Furthermore, activation of FXR and GPBAR1 have also been shown lead to suppression of NF-κB (45). Altogether, our findings provide new insights into the mechanisms of the anti-inflammatory effect of LcS. An illustrated overview of the proposed mechanism of LcS in IBD is presented in Figure 7.

In conclusion, we have identified LcS as a potential anti-inflammatory probiotic for the treatment of IBD. LcS treatment ameliorated disease manifestation in DSS-induced mice, restored intestine epithelial barrier function, and improved dysbiotic conditions. LcS modulated bile acid metabolism and suppressed the pro-inflammatory response. Mechanistically, LcS stabilized p-IκBα and suppressed activated NF-κB signaling. Our study provides evidence of the beneficial effects of LcS in IBD, as well as its regulatory effects on the gut microbiota and bile acid metabolism.

## Data Availability Statement

The original contributions presented in the study are included in the article/Supplementary Material, further inquiries can be directed to the corresponding authors.

## Ethics Statement

The animal study was reviewed and approved by the Animal Subjects Ethics Sub-Committee (ASESC) of The Hong Kong Polytechnic University and conducted in accordance with the Institutional Guidelines and Animal Ordinance of the Department of Health, Hong Kong SAR, China.

## Author Contributions

WC-ST and Y-WK: conceptualization. W-YW: formal analysis. W-YW, BC, T-TS, MM-LL, C-OC, C-TC, and DK-WM: investigation. W-YW, BC, and WC-ST: writing—original draft preparation. W-YW, BC, Y-WK, and WC-ST: writing—review and editing. All authors read and approved the submitted version.

## Conflict of Interest

The authors declare that the research was conducted in the absence of any commercial or financial relationships that could be construed as a potential conflict of interest.

## Publisher’s Note

All claims expressed in this article are solely those of the authors and do not necessarily represent those of their affiliated organizations, or those of the publisher, the editors and the reviewers. Any product that may be evaluated in this article, or claim that may be made by its manufacturer, is not guaranteed or endorsed by the publisher.
