# Supplementary material for: Lactobacillus casei Strain Shirota Ameliorates Dextran Sulfate Sodium-Induced Colitis in Mice by Increasing Taurine-Conjugated Bile Acids and Inhibiting NF-κB Signaling via Stabilization of IκBα
Source: Front Nutr. 2022 Apr 21;9:816836. doi: 10.3389/fnut.2022.816836 (PMC9069136; doi:10.3389/fnut.2022.816836)
Supplement: Supplementary file 1 [file Table_1.DOCX]

**Table S1.** Optimized MRM parameters and elution time for quantification of bile acids and conjugated bile acids

| Standard | Bile acid abbreviation | Type | Retention time (min) | MRM transitions (m/z) | | | Dwell time (msec) | Declustering potential | Entrance potential | Collision energy (eV) | Collision cell exit potential | Internal standard |
| --- | --- | --- | --- | --- | --- | --- | --- | --- | --- | --- | --- | --- |
| Hyodeoxycholate | HDCA | 2° | 17.15 | 391.4 | → | 391.4 | 50 | -160 | -10 | -10 | -25 | Ursodeoxycholate-D_4_ |
| Ursodeoxycholate | UDCA | 2° | 17.01 | 391.4 | → | 391.4 | 50 | -160 | -10 | -10 | -25 |  |
| Ursodeoxycholate-D_4_ | - | - | 16.98 | 395.3 | → | 395.3 | 50 | -150 | -10 | -19 | -10 |  |
| Chenodeoxycholate | CDCA | 1° | 19.01 | 391.4 | → | 391.4 | 50 | -160 | -10 | -10 | -25 | Deoxycholate-D_4_ |
| Deoxycholate | DCA | 2° | 19.17 | 391.4 | → | 391.4 | 50 | -160 | -10 | -10 | -25 |  |
| Lithocholate | LCA | 2° | 20.23 | 375.3 | → | 375.3 | 100 | -120 | -10 | -20 | -15 |  |
| Deoxycholate-D_4_ | - | - | 19.17 | 395.3 | → | 395.3 | 50 | -130 | -10 | -53 | -18 |  |
| α-Muricholate | α-MCA | 1° | 14.47 | 407.3 | → | 407.3 | 50 | -170 | -10 | -15 | -25 | Cholate-D_4_ |
| β-Muricholate | β-MCA | 1° | 15.01 | 407.3 | → | 407.3 | 50 | -170 | -10 | -15 | -25 |  |
| Cholate | CA | 1° | 16.67 | 407.3 | → | 407.3 | 50 | -170 | -10 | -15 | -25 |  |
| Cholate-D_4_ | - | - | 16.65 | 411.3 | → | 411.3 | 50 | -170 | -10 | -15 | -25 |  |
| Glycoursodeoxycholate | GUDCA | 2° | 14.04 | 448.4 | → | 74.1 | 50 | -140 | -10 | -80 | -20 | Glycoursodeoxycholate-D_4_ |
| Glycohyodeoxycholate | GHDCA | 2° | 14.36 | 448.4 | → | 74.1 | 50 | -140 | -10 | -80 | -20 |  |
| Tauroursodeoxycholate | TUDCA | 2° | 10.36 | 498.0 | → | 80.0 | 50 | -134 | -10 | -130 | -12 |  |
| Taurohyodeoxycholate | THDCA | 2° | 10.07 | 498.0 | → | 80.0 | 50 | -134 | -10 | -130 | -12 |  |
| Glycoursodeoxycholate-D_4_ | - | - | 13.94 | 452.4 | → | 74.1 | 50 | -140 | -80 | -10 | -10 |  |
| Glycochenodeoxycholate | GCDCA | 1° | 17.03 | 448.4 | → | 74.1 | 50 | -140 | -10 | -80 | -20 | Glycochenodeoxycholate-D_4_ |
| Glycodeoxycholate | GDCA | 2° | 17.46 | 448.4 | → | 74.1 | 50 | -140 | -10 | -80 | -20 |  |
| Taurochenodeoxycholate | TCDCA | 1° | 15.04 | 498.0 | → | 80.0 | 50 | -134 | -10 | -130 | -12 |  |
| Taurodeoxycholate | TDCA | 2° | 15.63 | 498.0 | → | 80.0 | 50 | -134 | -10 | -130 | -12 |  |
| Glycochenodeoxycholate-D_4_ | - | - | 16.99 | 452.4 | → | 74.1 | 50 | -140 | -80 | -10 | -10 |  |
| Glycocholate | GCA | 1° | 14.18 | 464.3 | → | 74.1 | 50 | -60 | -10 | -90 | -10 | Glycocholate-D_4_ |
| Taurocholate | TCA | 1° | 10.73 | 514.0 | → | 80.0 | 50 | -170 | -10 | -139 | -8 |  |
| Glycocholate -D_4_ | - | - | 14.12 | 468.0 | → | 74.1 | 50 | -175 | -10 | -85 | -35 |  |
